# Recent advances in the mechanisms, management, and nanomaterial-based strategies for root resorption

**DOI:** 10.1186/s11671-026-05113-4

**Published:** 2026-11-03

**Authors:** Lin Zhang, Yuhang Wei, Zhouqiao Xie, Hanwei Yu, Xutong Zhu, Siyuan Li, Xuejun Ge, Yuting Niu, Nan Jiang

**Affiliations:** 1 Peking University Hospital of Stomatology Sanya Division (Sanya Stomatology Center), No. 9, Linwang South Road, Haitang District, Sanya City, 572013 Hainan Province PR China; 2 Central Laboratory, Peking University School and Hospital of Stomatology & Beijing Key Laboratory of Intelligent Biomanufacturing and Regeneration of Craniofacial Tissues & National Center of Stomatology & National Clinical Research Center for Oral Diseases & National Engineering Research Center of Oral Biomaterials and Digital Medical Devices, No. 22, Zhongguancun South Avenue, Haidian District, Beijing, 100081 PR China; 3 Shanxi Province Key Laboratory of Oral Diseases Prevention and New Materials, Shanxi Medical University School and Hospital of Stomatology, No. 63 Xinjian South Road, Taiyuan, 030001 Shanxi PR China; 4 Department of Prosthodontics, Peking University School and Hospital of Stomatology, No. 22, Zhongguancun South Avenue, Haidian District, Beijing, 100081 PR China; 5 Central Laboratory, Tianjin Stomatological Hospital, School of Medicine, Nankai University & Tianjin Key Laboratory of Oral and Maxillofacial Function Reconstruction & Stomatology Institute of Nankai University, Tianjin, 300041 PR China

**Keywords:** Root resorption, Odontoclastogenesis, Nanomaterials, Targeted drug delivery, Dental tissue regeneration

## Abstract

Root resorption (RR) involves the loss of mineralized root tissues and may compromise tooth integrity and long-term retention. Internal and external RR arise in different pulpal and periodontal microenvironments and require etiology-specific management. Although conventional treatment can arrest many lesions, persistent infection, complex resorptive defects, and limited control of inflammatory or clastic-cell activity remain therapeutic challenges. This narrative review summarizes RR classification, clinical management, and key mechanisms, including protective-tissue disruption, receptor activator of nuclear factor-κB ligand (RANKL)-dependent clastic-cell activation, inflammatory signaling, and mechanotransduction, and evaluates nanomaterial-based approaches by function. Potential benefits are weighed against cytotoxicity, local retention, effects on physiological remodeling, and the risk of inappropriate bone formation or ankylosis. Most current evidence remains preclinical, and substantial challenges related to efficacy, safety, targeting, manufacturing, and clinical validation must be addressed before clinical translation. Whether nanomaterials developed for bone resorption can be adapted to promote cementum-like repair of the resorbed root surface remains a hypothesis requiring RR- and cementum-specific validation.

## Introduction

### Overview of root resorption

Root resorption (RR) is characterized by the progressive removal of mineralized dental tissues through the activity of odontoclasts [1]. Under physiological conditions, the root surface is protected by specialized mineralized and unmineralized tissues that prevent clastic cells from attaching to the root. When these protective structures are disrupted by trauma, infection, orthodontic forces, or other pathological stimuli, the underlying mineralized matrix becomes exposed. Injury affecting the odontoblast layer and predentin mainly predisposes to internal root resorption (IRR), whereas destruction of the precementum and periodontal ligament primarily results in external root resorption (ERR). The ensuing inflammatory microenvironment stimulates the production of cytokines and chemokines that recruit and activate odontoclasts, ultimately leading to progressive degradation of root tissues [1]. Root resorption serves distinct biological roles depending on the stage of dentition. During the primary dentition, physiological resorption is an essential developmental process that permits exfoliation of deciduous teeth and eruption of their permanent successors. By contrast, resorption affecting permanent teeth is considered a pathological event. Root resorption in permanent teeth is broadly classified as internal root resorption or external root resorption according to the site of lesion initiation. Because permanent dental tissues possess limited regenerative capacity, these lesions are generally progressive and require timely diagnosis, appropriate intervention, or long-term clinical surveillance [2].

Conventional local drug-delivery approaches for RR may be limited by insufficient retention, uncontrolled release, and restricted access to anatomically complex lesions. In periodontal applications, gingival crevicular fluid and salivary clearance can shorten local residence time, whereas intracanal delivery is constrained by the complex anatomy of the root canal system and limited penetration into inaccessible regions [3, 4]. Systemic administration may further limit drug exposure at the lesion while increasing unnecessary systemic exposure [5]. These limitations provide a rationale for delivery platforms capable of improving local retention, controlled release, and site-specific drug availability.

Growing advances in nanomaterial engineering have expanded the potential applications of nanotechnology in dentistry. Owing to their tunable physicochemical properties, high surface-area-to-volume ratios, and capacity for surface functionalization, nanomaterial-based platforms have been investigated for localized and controlled drug delivery, antimicrobial therapy, and tissue-regenerative applications in oral and dental diseases [6–8]. In endodontics, nanoparticles have been incorporated into irrigants, intracanal medicaments, sealers, and other delivery systems to improve antimicrobial activity against persistent root-canal biofilms [9, 10]. Nanostructured materials have also been investigated as drug-delivery vehicles and regenerative scaffolds capable of supporting the delivery of bioactive molecules and dental tissue regeneration [7, 11]. However, most of these applications have been evaluated in general dental, endodontic, or regenerative models rather than in RR-specific models. Therefore, their potential relevance to RR should be considered indirect or extrapolated evidence until validated in appropriate root-resorption models. In the context of RR, these properties may be relevant to several therapeutic needs, including microbial control in infection-associated lesions, localized delivery of anti-inflammatory or anti-resorptive agents, and support for the repair of resorptive defects. However, direct evidence for these applications in RR-specific models remains limited, and much of the current rationale is derived from endodontic, periodontal, regenerative, or bone-related studies.

Accordingly, this review integrates the pathophysiological and clinical heterogeneity of RR with subtype-specific therapeutic needs and the corresponding functions of nanomaterial-based strategies. Particular emphasis is placed on distinguishing direct RR evidence from indirect dental evidence and evidence extrapolated from periodontal or bone models, while critically evaluating indication-specific efficacy, safety, and barriers to clinical translation. By linking RR etiology and therapeutic requirements to the level of evidence supporting individual nanomaterial-based approaches, this review aims to provide a more clinically and biologically grounded framework for future RR-specific nanotherapeutic research.

### Literature search and review methodology

This narrative review was conducted using a structured literature search of PubMed: https://pubmed.ncbi.nlm.nih.gov/. The search strategy was designed to capture four complementary evidence domains: (1) the pathophysiology and molecular mechanisms of RR; (2) studies directly investigating nanomaterial-based approaches in RR; (3) indirect evidence from endodontic, periodontal, cementum-related, and other relevant dental models; and (4) evidence concerning the safety, cytotoxicity, biocompatibility, and translational limitations of nanomaterials.

Search terms included combinations of RR-related terms, such as “root resorption,” “internal root resorption,” “external root resorption,” “external inflammatory root resorption,” “external cervical resorption,” “orthodontically induced root resorption,” and “odontoclast,” together with mechanistic terms including RANKL, OPG, NFATc1, macrophage, osteoclast, mechanotransduction, Piezo1, YAP/TAZ, and SphK1. Nanomaterial-related terms included “nanomaterial,” “nanoparticle,” “nano-hydroxyapatite,” “silver nanoparticle,” “graphene oxide,” “mesoporous silica,” “chitosan,” “tetrahedral framework nucleic acid,” and “exosome.” Additional searches incorporated terms related to antimicrobial activity, drug delivery, mineralization, periodontal regeneration, toxicity, cytotoxicity, biocompatibility, and adverse effects.

**Fig. 1:**
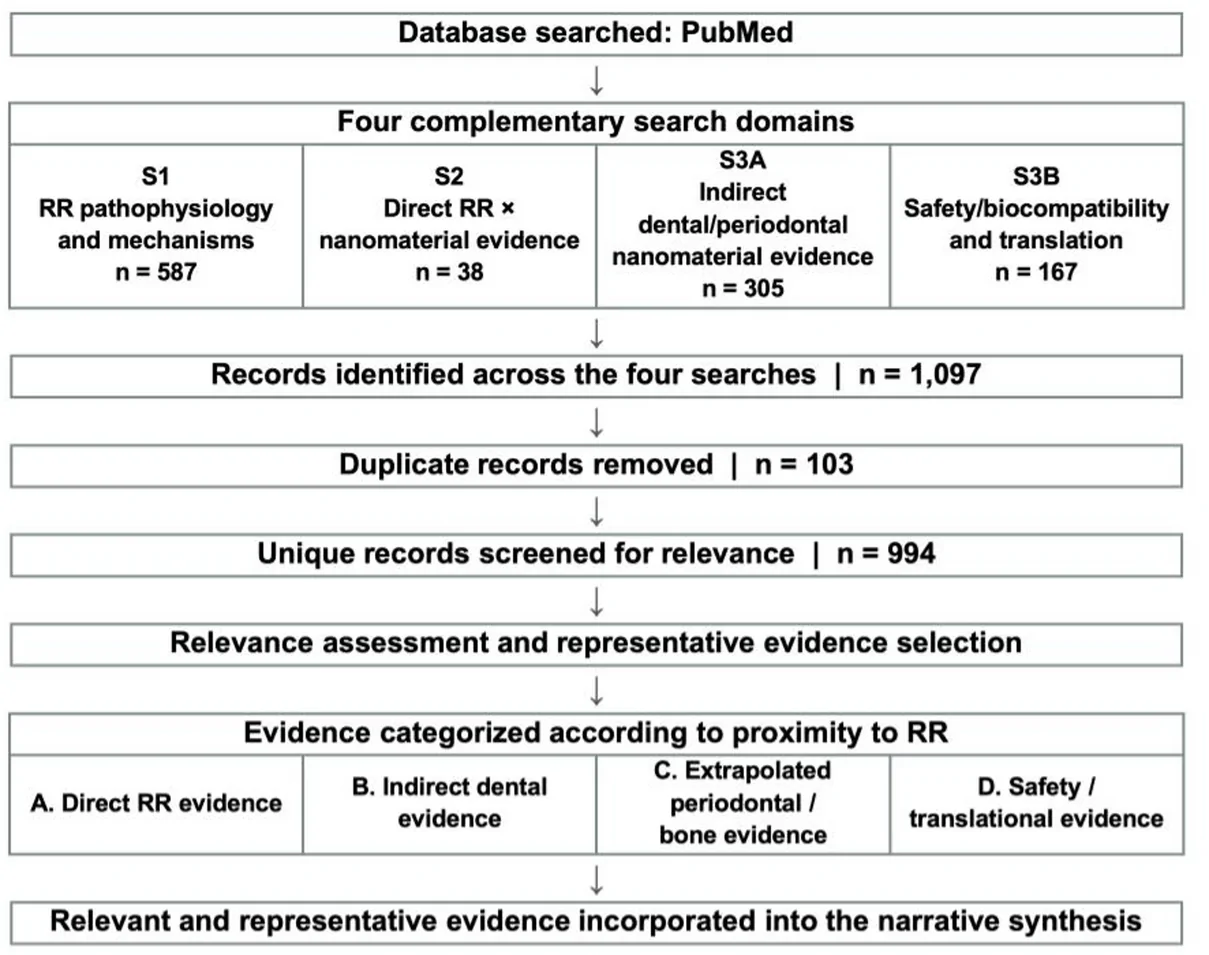
Structured literature search and evidence-selection process for the narrative review

The four complementary searches initially identified 1,097 records. After removal of 103 duplicate records, 994 unique records were screened for relevance (Fig. 1). Studies were considered eligible when they provided evidence relevant to RR pathophysiology, nanomaterial-based intervention, safety, or clinical translation. Because direct RR-specific evidence for several nanomaterial-based strategies remains limited, relevant endodontic, periodontal, cementum-related, and bone-resorption studies were also considered when they provided a clear mechanistic or translational context for RR. Studies were excluded when they were unrelated to RR or RR-relevant biological mechanisms, investigated materials outside the scope of this review, or provided no relevant mechanistic, therapeutic, safety, or translational information.

During evidence synthesis, studies were interpreted according to their proximity to RR and were categorized as direct RR evidence, indirect dental evidence, extrapolated periodontal or bone evidence, or safety and translational evidence. Greater weight was given to studies directly investigating pathological RR and to evidence most closely aligned with the specific therapeutic requirements of individual RR subtypes. Indirect or extrapolated findings were explicitly identified as such and were not interpreted as evidence of established efficacy in RR. Studies addressing adverse effects, cytotoxicity, biocompatibility, and translational limitations were also considered to provide a balanced assessment of nanomaterial-based strategies.

As this study was designed as a narrative rather than a systematic review, no formal meta-analysis or quantitative risk-of-bias assessment was performed. The final literature search was conducted on September 29, 2026.

## Classification and clinical management of root resorption

Root resorption is broadly categorized according to the anatomical site at which the lesion originates [1, 2]. Internal root resorption (IRR) develops within the root canal system and includes inflammatory and replacement forms. Inflammatory internal resorption is associated with persistent pulpal inflammation, which promotes clastic cell activity and progressive loss of intraradicular dentin. In contrast, internal replacement resorption is characterized by the deposition of bone- or cementum-like hard tissue within the resorptive area [12].

**Fig. 2:**
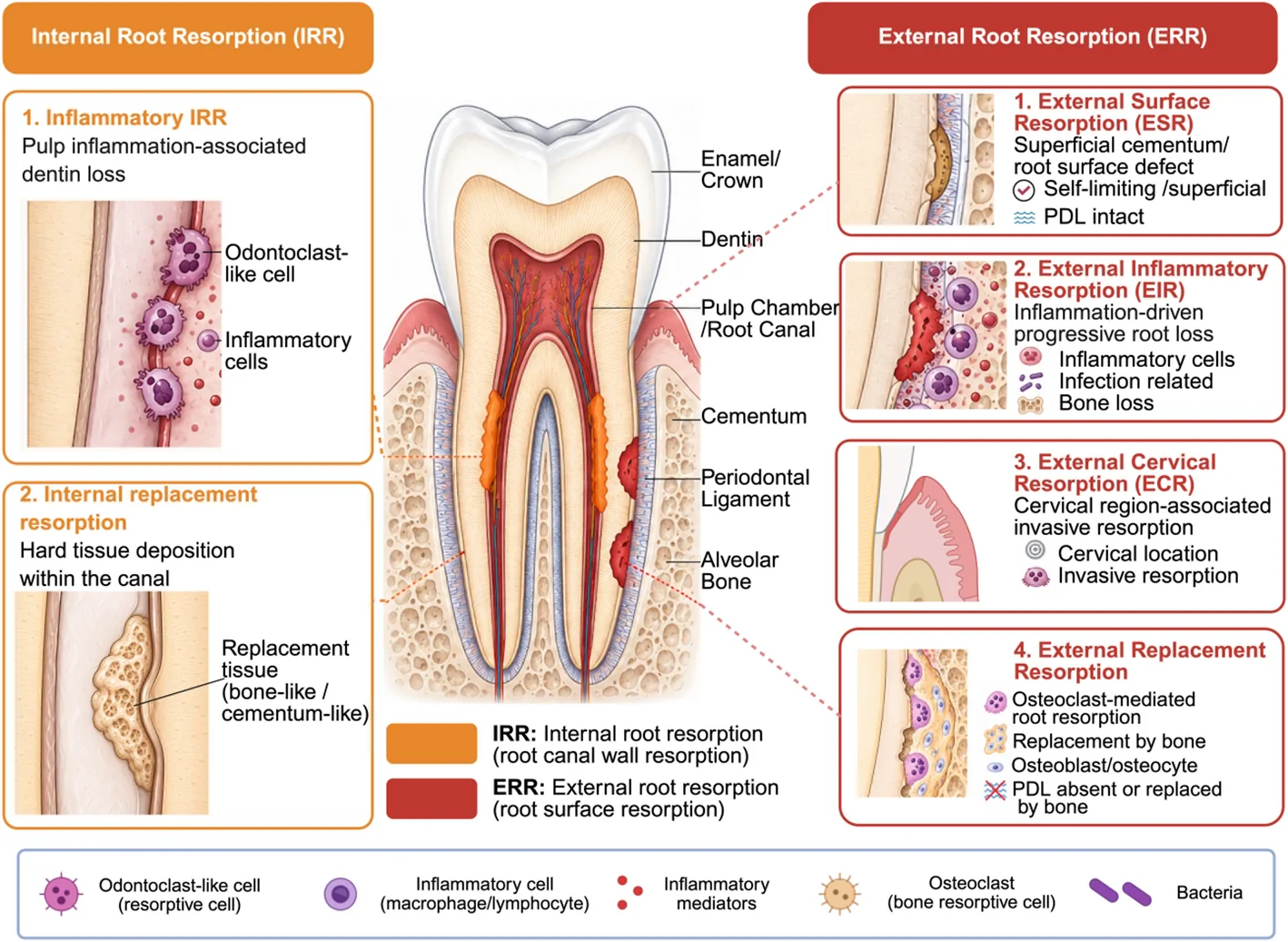
Schematic overview of the major patterns of internal and external root resorption. Internal root resorption (IRR) originates from the pulp–dentin interface and includes inflammatory and replacement forms, whereas external root resorption (ERR) originates from the external root surface and includes external surface resorption (ESR), external inflammatory resorption (EIR), external cervical resorption (ECR), and external replacement resorption. Created in https://BioRender.com

External root resorption (ERR) originates from the external root surface and includes external surface resorption (ESR), external inflammatory resorption (EIR), external cervical resorption (ECR), and external replacement resorption. These subtypes differ in their anatomical locations and pathological characteristics. ESR typically involves limited superficial root surface resorption, whereas EIR is characterized by inflammation-driven progressive root tissue destruction, and ECR represents an invasive resorptive process primarily affecting the cervical region of the tooth [1, 2]. External replacement resorption involves progressive replacement of resorbed root tissue by bone, accompanied by disruption or loss of the normal periodontal ligament interface (Fig. 2).

### Internal root resorption (IRR)

Internal root resorption (IRR) originates within the root canal system and involves clastic-cell-mediated loss of intraradicular dentin. It is commonly classified into inflammatory and replacement forms according to the tissue changes within the resorptive defect [2, 12]. Cone-beam computed tomography (CBCT) enables three-dimensional assessment of the location and extent of IRR and facilitates the detection of root perforation, thereby supporting diagnosis and treatment planning [13, 14].

For inflammatory IRR, management primarily aims to eliminate inflamed or infected intracanal tissue and the stimulus sustaining clastic activity, followed by effective disinfection and sealing of the resorptive defect. Nonsurgical root canal treatment remains the principal approach for restorable teeth, whereas perforating or inaccessible lesions may require additional surgical management, and extraction may be necessary when extensive structural destruction precludes predictable tooth preservation. However, the irregular geometry of resorptive cavities can complicate instrumentation, disinfection, and adequate filling of the defect [15]. These therapeutic challenges highlight potential opportunities for approaches that provide enhanced antimicrobial activity, localized and sustained drug delivery, and bioactive support for tissue repair. Accordingly, nanomaterial-based strategies addressing these functions may be relevant to infection-associated IRR; however, direct evidence from IRR-specific experimental or clinical studies remains limited.

### External root resorption (ERR)

#### External surface resorption (ESR)

External surface resorption (ESR) is a localized, non-infective and generally self-limiting form of root resorption associated with limited injury to the external root surface and periodontal ligament [16]. Accordingly, management primarily focuses on identifying and eliminating the underlying mechanical factor, and removing the causative stimulus [16–18]. When the periodontal ligament and reparative capacity of the root surface remain intact, the resorbed area may subsequently undergo spontaneous repair through deposition of new cementum [19, 20]. Therefore, ESR usually does not require additional pharmacological intervention after effective removal of the causative stimulus. Given its predominantly self-limiting nature and intrinsic capacity for cementum repair, the therapeutic rationale for nanomaterial-based intervention in uncomplicated ESR is currently limited. Nanotechnology-based approaches would be more relevant only if future studies identify specific indications in which spontaneous repair is impaired or resorption persists despite removal of the initiating stimulus.

#### External inflammatory resorption (EIR)

**Fig. 3:**
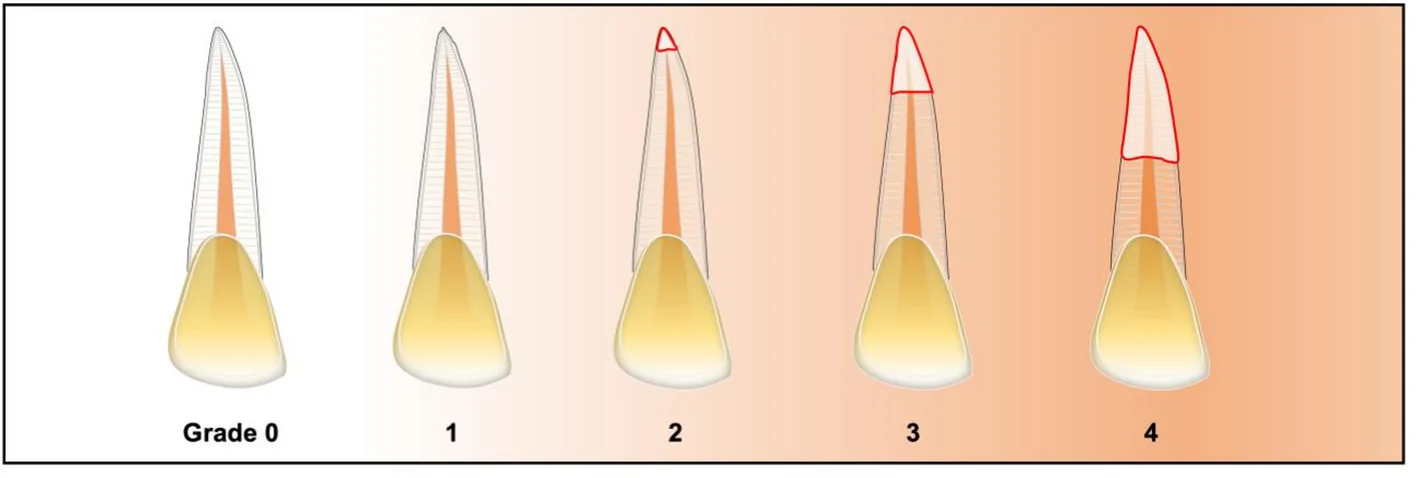
Levander–Malmgren grading system for orthodontically induced external apical root resorption. Grade 0, no resorption; Grade 1, irregular apical contour without appreciable root shortening; Grade 2, minor apical resorption with root shortening of < 2 mm; Grade 3, root loss of 2 mm to one-third of the original root length; and Grade 4, severe resorption exceeding one-third of the original root length

External inflammatory resorption (EIR) may arise following dental trauma, pulpal infection, or excessive orthodontic loading, although its biological drivers and therapeutic requirements differ according to etiology. For orthodontically induced external apical root resorption, severity can be assessed using the Levander–Malmgren grading system, ranging from Grade 0 (no resorption) to Grade 4 (severe root loss exceeding one-third of the original root length) [21, 22] (Fig. 3). In infection- or trauma-associated EIR, microbial products and the resulting inflammatory response provide a sustained stimulus for clastic-cell recruitment and activation. Management therefore primarily aims to eliminate the microbial source through effective root-canal disinfection and thereby arrest inflammation-driven resorption [23–27]. However, the complex anatomy of the root canal system and resorptive defects can restrict antimicrobial penetration and local drug retention, potentially limiting effective control of the inflammatory stimulus. These limitations provide a rationale for localized delivery systems capable of improving antimicrobial activity, sustained drug exposure, and, where appropriate, modulation of inflammation and clastic-cell activity.

Orthodontically induced inflammatory root resorption (OIRR) differs from infection-associated EIR in that it primarily represents a mechanically induced sterile inflammatory response associated with orthodontic loading. Current clinical management relies mainly on modification or interruption of orthodontic force and subsequent monitoring, whereas pharmacological strategies for directly suppressing inflammation or odontoclast activity have not been established as routine clinical treatments [28]. Experimental anti-inflammatory and anti-resorptive strategies have attenuated OIRR in preclinical models, including approaches targeting macrophage polarization, tumor necrosis factor-alpha (TNF-α) signaling, and 12/15-lipoxygenase activity [29, 30]. Emerging molecular approaches involving non-coding RNAs and other mechanoinflammatory pathways are also under investigation but remain predominantly preclinical [28, 31, 32]. Accordingly, the principal therapeutic opportunity for nanomaterial-based strategies in OIRR lies not in antimicrobial treatment, but in the localized and controlled delivery of agents capable of modulating excessive inflammatory and odontoclastogenic signaling while minimizing interference with physiological periodontal and alveolar-bone remodeling.

#### External replacement resorption

External replacement resorption is characterized by progressive resorption of the root and its replacement by alveolar bone, typically following severe damage to or loss of the viable PDL. It is closely associated with dentoalveolar ankylosis and is a well-recognized complication of severe dental trauma, particularly avulsion followed by delayed replantation [33, 34]. Unlike infection-driven inflammatory resorption, replacement resorption primarily reflects loss of the functional PDL and subsequent incorporation of the ankylosed root into physiological alveolar-bone remodeling [33, 35]. Once established, the process cannot currently be predictably arrested or reversed, and management therefore focuses on monitoring progression and preserving alveolar ridge dimensions and future rehabilitation options, particularly in growing patients [36, 37].

#### External cervical resorption (ECR)

External cervical resorption (ECR) is a distinct form of ERR that originates in the cervical region of the root and may progress extensively before becoming clinically apparent [38]. Its etiology and biological drivers remain incompletely understood, and management is therefore guided primarily by lesion location, extent, accessibility, and pulpal involvement. Treatment generally aims to arrest the resorptive process, remove resorptive tissue where feasible, and restore the resulting defect, whereas advanced or inaccessible lesions may require monitoring or extraction [38–40]. The irregular geometry and variable accessibility of ECR lesions can complicate complete debridement and durable restoration, representing an important therapeutic limitation. However, because the biological drivers and optimal molecular targets of ECR remain incompletely defined, the rationale for specific nanotherapeutic interventions remains largely theoretical, and direct evidence supporting nanomaterial-based interventions specifically for ECR remains very limited.

Taken together, the therapeutic requirements of RR differ substantially according to lesion etiology. Infection-associated IRR and EIR primarily require effective microbial control together with suppression of inflammation-driven clastic activity, whereas OIRR represents a mechanically induced sterile inflammatory process in which modulation of inflammatory and odontoclastogenic signaling is more relevant than antimicrobial intervention. ESR is generally self-limiting after removal of the causative stimulus and therefore provides a limited rationale for additional pharmacological intervention. In contrast, external replacement resorption is associated with severe PDL damage and ankylosis, followed by progressive replacement of the root by alveolar bone [33–35].

These subtype-specific differences highlight the importance of understanding the biological mechanisms that initiate and sustain pathological root resorption. Although the initiating stimuli and clinical manifestations vary among RR subtypes, several shared pathways involving disruption of protective root tissues, inflammatory signaling, and clastic-cell activation contribute to disease progression. The following section therefore examines these mechanisms and identifies potential molecular targets relevant to the development of nanomaterial-based therapeutic strategies.

## Pathophysiological basis of root resorption

### Protective tissues of the root

The tooth root is composed of dentin as the main body of the hard root tissue, the surrounding cementum and periodontal ligament, and the pulp cavity. Throughout normal tooth function, occlusal loading and routine mechanical stimulation are transmitted from the crown to the root without causing resorption. This resistance is largely attributable to specialized unmineralized protective layers that cover both the internal and external root surfaces. The odontoblast layer and predentin shield the root canal wall, whereas the precementum and periodontal ligament protect the external root surface. By preventing direct contact between clastic cells and mineralized root tissues, these unmineralized layers contribute to the maintenance of root homeostasis and resistance to pathological resorption [41–43]. Studies have shown that when predentin is treated with guanidine hydrochloride to remove its organic components, the exposed mineralized dentin surface promotes odontoclast aggregation, highlighting the essential role of this organic layer in resisting resorption [44].

Under pathological conditions, however, this protective mechanism can fail. For instance, excessive orthodontic or occlusal forces can compress cementoblasts against the root surface. This mechanical overload triggers apoptosis and functional impairment of cementoblasts [45], which in turn diminishes the replenishment of the protective outer layer and contributes to the breakdown of the root’s natural defense system [1]. Similarly, infection or traumatic injury may cause ischemic and inflammatory damage to pulpal or periodontal tissues, disrupting the local balance between root protection and resorption. Mechanical or inflammatory disruption of predentin internally or precementum and the periodontal ligament externally exposes the underlying mineralized root surface to the surrounding microenvironment. This injury activates local resident cells, including macrophages, periodontal ligament stem cells, periodontal ligament fibroblasts, and cementoblasts, which secrete inflammatory cytokines such as interleukin-1β (IL-1β), interleukin-6 (IL-6), and TNF-α. These mediators establish a pro-resorptive microenvironment by enhancing RANKL-dependent recruitment, differentiation, and activation of clastic-cell precursors [2, 45, 46]. Importantly, even after RR has been initiated, residual protective tissues may still exert a partial inhibitory effect, helping to limit the progression of resorption [46].

### Activation and regulation of root-resorbing clastic cells

**Fig. 4:**
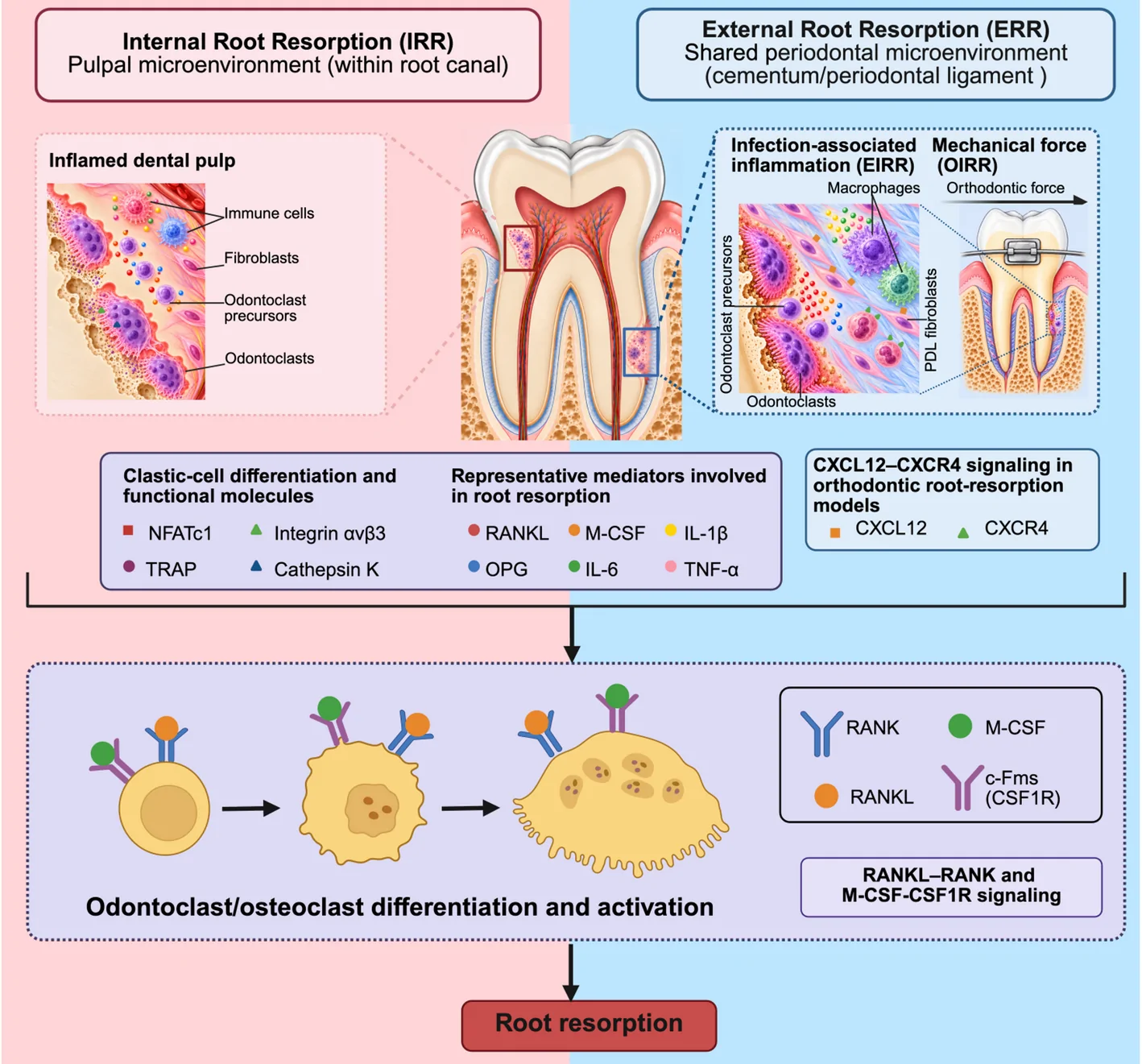
Distinct microenvironments and shared molecular mechanisms of internal and external root resorption. Schematic illustration of the anatomical microenvironments and molecular mechanisms underlying internal root resorption (IRR) and external root resorption (ERR). IRR originates within the root canal system, where pulpal inflammation promotes odontoclast activation, whereas ERR occurs at the external root surface and is associated with inflammatory or mechanical stimuli. External inflammatory root resorption (EIRR) and orthodontically induced root resorption (OIRR) represent distinct initiating conditions. Despite these differences, both IRR and ERR involve shared clastic-cell regulatory pathways, including RANKL–RANK, M-CSF–CSF1R, and downstream NFATc1 signaling. Additional mediators include TRAP, cathepsin K, integrin αvβ3, OPG, and inflammatory cytokines (IL-1β, IL-6, and TNF-α). CXCL12–CXCR4 signaling is also implicated in experimental OIRR. Created in https://BioRender.com

Odontoclasts are tooth-resorbing clastic cells that share major developmental and functional features with bone-resorbing osteoclasts. In this review, “odontoclast” is used when referring specifically to root resorption, “osteoclast” when describing bone-resorption studies, and “clastic cells” when discussing mechanisms shared by both cell populations. Evidence derived from osteoclast or bone models is explicitly identified as extrapolated when applied to RR. Pulpal or periodontal infection and excessive orthodontic loading can establish local inflammatory microenvironments that promote the recruitment, differentiation, and functional activation of clastic-cell precursors [47, 48]. These responses are coordinated through interconnected inflammatory, mechanosensitive, and clastic-cell differentiation pathways (Fig. 4).

Clastic-cell differentiation is principally regulated by macrophage colony-stimulating factor (M-CSF) and receptor activator of nuclear factor-κB ligand (RANKL) [49–51]. Binding of RANKL to its receptor RANK (receptor activator of nuclear factor-κB) on mononuclear precursors activates downstream signaling, including nuclear factor of activated T cells, cytoplasmic 1 (NFATc1), thereby promoting their differentiation, fusion, and functional activation. Mature multinucleated clastic cells subsequently adhere to mineralized surfaces through molecules such as integrin αvβ3 and express resorption-associated enzymes, including tartrate-resistant acid phosphatase (TRAP) and cathepsin K [52, 53]. The balance between RANKL and its soluble decoy receptor osteoprotegerin (OPG) is therefore a major determinant of odontoclastogenesis and resorptive activity. OPG sequesters RANKL and limits RANK activation, whereas increased RANKL availability favors clastic-cell differentiation and activation. Within dental and periodontal tissues, inflammatory mediators such as IL-1β, IL-6, and TNF-α can shift the local RANKL/OPG balance toward a pro-resorptive state, thereby enhancing odontoclastogenesis and root-tissue degradation [52, 54–58]. Consistent with this mechanism, compressed periodontal ligament (PDL) cells from patients with severe orthodontically induced external apical root resorption showed increased RANKL, decreased OPG, and enhanced osteoclastogenesis compared with cells from patients without resorption [56].

Macrophages provide an additional link between mechanically induced inflammation and root resorption. Although macrophage activation occurs along a continuous spectrum, pro-inflammatory M1-like states are generally associated with a microenvironment that favors clastic-cell formation, whereas M2-like states are more closely associated with inflammation resolution and tissue repair. In an experimental orthodontic root-resorption model, an increased M1/M2 macrophage ratio was associated with more severe root resorption, whereas force removal or interventions that reduced the M1/M2 ratio attenuated resorption [29]. Subsequent work further demonstrated that force-induced CXCL12/CXCR4 signaling recruits inflammatory monocytes and modulates the M1/M2 balance in periodontal tissues, providing a mechanistic link between mechanical loading, macrophage responses, and orthodontic root resorption [59].

Mechanotransduction provides a further mechanism through which orthodontic force is coupled to inflammatory and clastic-cell responses. Among the mechanosensitive ion channels expressed in periodontal tissues, PIEZO1 has emerged as an important mediator of force sensing in PDL cells. Earlier studies of orthodontic tooth movement demonstrated that PIEZO1 responds to mechanical loading and participates in the regulation of periodontal and alveolar-bone remodeling [60]. More importantly, recent direct evidence from orthodontic root-resorption models indicates that PIEZO1 contributes to pathological root resorption. Mechanical loading induces IL-6-dependent PIEZO1 activation in PDL cells, which enhances CXCL12/CXCR4-mediated recruitment of inflammatory monocytes and promotes M1-associated root resorption [61]. These findings establish a mechanistic connection between force sensing by PDL cells, inflammatory-cell recruitment, and the development of orthodontic root resorption.

YAP and TAZ represent another force-responsive signaling system that may connect mechanical sensing with periodontal remodeling. Recent evidence indicates that orthodontic tension activates PIEZO1-mediated Ca²⁺ influx in periodontal ligament stem cells, promoting YAP/TAZ nuclear accumulation and altering osteogenic differentiation and the OPG/RANKL balance [62]. However, this evidence derives primarily from models of orthodontic alveolar-bone remodeling rather than direct assessment of root resorption. The PIEZO1–Ca²⁺–YAP/TAZ axis should therefore be considered a mechanistically relevant pathway with potential implications for OIRR, but its specific role in pathological root resorption requires further direct validation.

In addition to these mechanosensitive pathways, recent evidence has identified SphK1-dependent mitophagy and mitochondrial transfer as an emerging mechanism of cementocyte–osteoclast communication during excessive orthodontic loading. Heavy orthodontic force increased SphK1 expression and PINK1/PARKIN-associated mitophagy in cementocytes, facilitating mitochondrial transfer to osteoclast precursors and enhancing their metabolic and resorptive activity [63]. Pharmacological or genetic inhibition of SphK1 suppressed these responses and attenuated orthodontically induced inflammatory root resorption. However, this mechanism is currently supported by a limited body of evidence and should therefore be regarded as an emerging pathway rather than an established central mechanism of OIRR. Independent validation in additional experimental systems will be necessary to establish its broader relevance.

Collectively, current evidence supports an integrated model in which orthodontic force is translated into root-resorptive activity through interconnected mechanosensitive, inflammatory, and clastic-cell regulatory pathways. The RANKL/RANK/OPG axis remains a central effector of clastic-cell differentiation, while macrophage responses and mechanosensitive signaling, particularly PIEZO1, link periodontal mechanical cues to inflammatory and clastic-cell responses. YAP/TAZ signaling provides an additional mechanistic link supported primarily by orthodontic periodontal and bone-remodeling studies, whereas SphK1-dependent mitophagy and mitochondrial transfer represent an emerging OIRR mechanism that requires further independent validation.

### Mechanisms of root-tissue degradation

Following exposure of mineralized dentin or cementum, differentiated odontoclasts adhere to the root surface through integrin-mediated interactions with matrix proteins [53]. Cytoskeletal reorganization subsequently establishes a sealed resorption compartment and ruffled border, within which proton secretion acidifies and demineralizes the mineralized matrix. Cathepsin K and matrix metalloproteinases then degrade the exposed organic matrix, allowing continued root-tissue resorption [64].

Importantly, strategies intended to promote mineralization or bone formation should not automatically be assumed to benefit all forms of RR, because preservation of a functional periodontal ligament is important for reducing the risk of ankylosis and replacement resorption. This consideration is particularly relevant when extrapolating regenerative or osteogenic findings from bone-related models to RR therapy. Together, the subtype-specific therapeutic requirements outlined in Sect.  2 and the molecular mechanisms discussed in Sect.  3 provide a framework for evaluating nanomaterial-based strategies, including antimicrobial platforms, localized drug-delivery systems, anti-inflammatory and anti-clastic approaches, and materials intended to support tissue repair.

## Applications of nanomaterials in root resorption

Root resorption is multifactorial, and current treatment remains limited to removing the causative factors and restricting further tissue destruction. Recent progress in nanomaterials has opened new options for localized drug delivery, repair of resorptive defects, and regeneration of the affected dental tissues.

### Overview of nanomaterial therapy

**Fig. 5:**
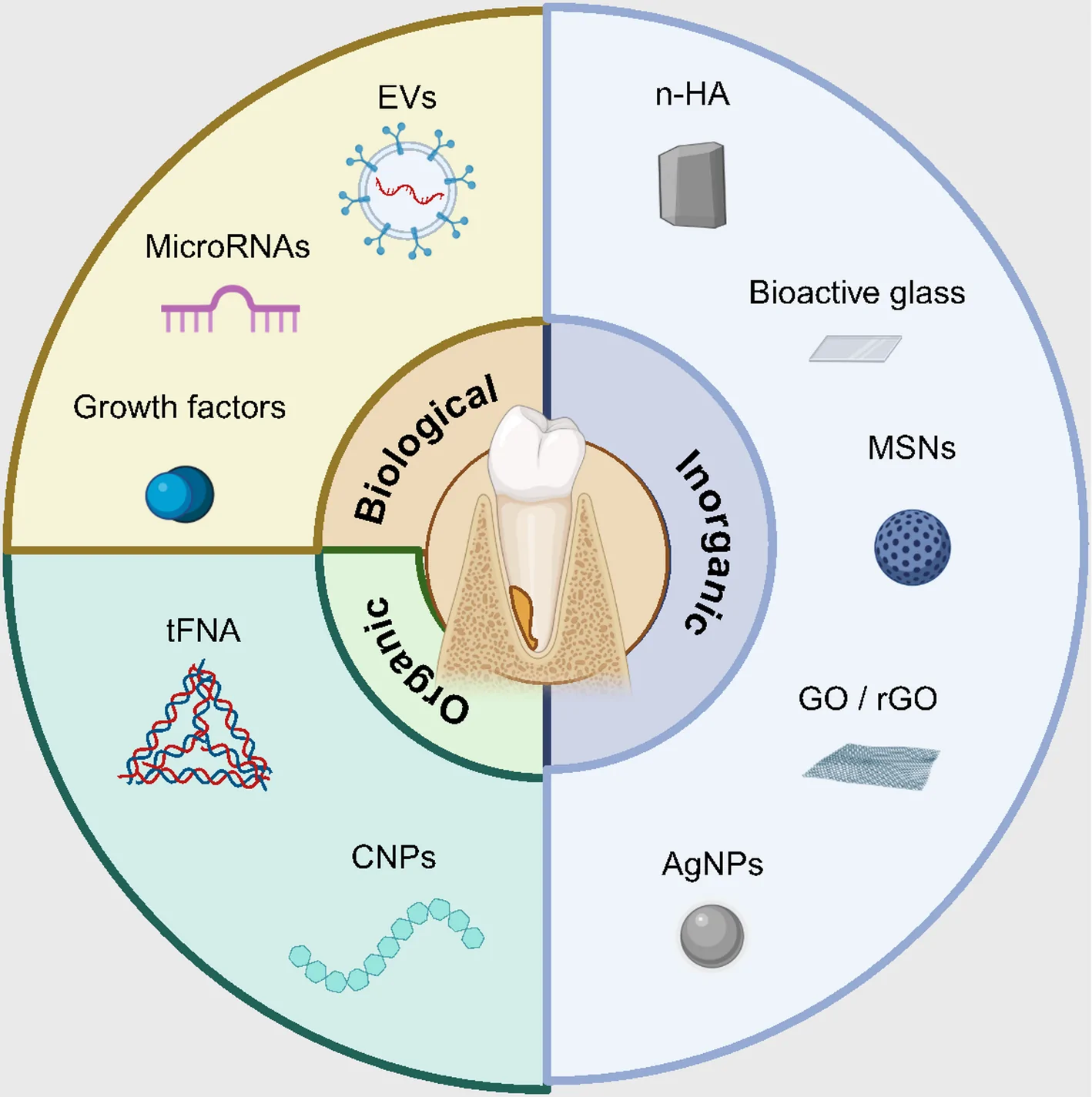
Classification of inorganic, organic, and biological nanoscale systems and therapeutic cargo for root resorption therapy. n-HA, nano-hydroxyapatite; MSNs, mesoporous silica nanoparticles; GO, graphene oxide; rGO, reduced graphene oxide; AgNPs, silver nanoparticles; tFNA, tetrahedral framework nucleic acids; CNPs, chitosan nanoparticles; EVs, extracellular vesicles. Created in https://BioRender.com

Nanomaterials are grouped into three classes. The inorganic class comprises metals, metal oxides, and carbon-based nanostructures. The organic class comprises polymeric and nucleic-acid-based materials such as chitosan nanoparticles and tetrahedral framework nucleic acids, and the biological nanoscale systems and therapeutic cargo group comprises cell-derived extracellular vesicles (EVs) together with the cargo molecules they deliver, such as microRNAs and growth factors or peptides. The delivery of these cargo molecules may be enhanced by vesicles, nanocarriers, or other local delivery systems. The grouping is practical rather than taxonomic, because hybrid nanocomposites may span more than one class (Fig. 5). Structurally, these materials occur as nanoparticles (spheres), nanorods, nanotubes, nanofibers, or nanosheets, each a distinct unit at the nanoscale (1–100 nm); cell-derived extracellular vesicles are an exception, being heterogeneous and extending beyond this range (Sect.  4.2.3.1).

Functionalized nanoparticles typically consist of a nanoscale core carrying therapeutic or diagnostic components, either embedded within the particle or immobilized on its surface. Drugs, peptides, antibodies, fluorophores, and other bioactive molecules can be attached to improve targeting, imaging, stability, or therapeutic performance [65], usually through bifunctional linkers that connect reactive groups on the particle to biocompatible polymers, targeting ligands, or imaging probes. Alternatively, the core can act as a scaffold for the assembly of additional organic or inorganic components, yielding multifunctional nanostructures [65]. At this scale, a high surface-to-volume ratio and altered surface chemistry give rise to behavior that differs substantially from that of the bulk material. These differences include enhanced chemical reactivity, tunable surface charge, improved molecular interactions, and material-dependent optical, electrical, or mechanical properties. Such features allow nanomaterials to support localized drug delivery, antimicrobial activity, modulation of inflammation, and tissue repair, and thus offer new options for the management of root resorption.

Building on established principles for the management of RR [40], this review divides nanomaterial-based strategies into preventive and therapeutic approaches. Preventive strategies aim to interrupt resorption at an early stage: antibacterial nanomaterials, or materials that suppress clastic-cell differentiation—used alone or as drug-loaded carriers—are delivered into the periodontal ligament and root canal, where they inhibit the differentiation and activity of clastic cells. By targeting the etiological factors and limiting progression, this approach has reduced resorption in preclinical models of orthodontically induced root resorption [66, 67], although treatment strategies for OIRR remain experimental [28]. The risk is not uniform across teeth: a meta-analysis found less orthodontically induced external root resorption in endodontically treated teeth than in their contralateral vital counterparts, indicating that root canal treatment does not by itself increase this risk [68]. Therapeutic strategies instead apply nanomaterials in regenerative dentistry to repair damaged root dentin, cementum, periodontal ligament, and alveolar bone, a process thought to involve signaling pathways associated with osteogenic and odontoblastic differentiation [69].

#### Physicochemical determinants of RR-relevant performance

Material class alone does not determine RR-relevant performance. Particle size and morphology, surface chemistry (including surface charge and hydrophobicity), and degradation behavior together govern cellular uptake, colloidal stability in physiological media, and how long a therapeutic concentration is maintained at the resorption site [70]. Delivery from the root canal or the periodontal sulcus is limited in practice by how far a material penetrates into the dentinal tubules and the periodontal ligament. Because these parameters vary widely within a single material class and are reported inconsistently, nanomaterials should not be treated as a homogeneous category when their potential for RR is assessed.

#### Targeting specificity

Targeting specificity is one of the least resolved problems in this field. An effective system should accumulate at the resorptive lesion while sparing healthy periodontal ligament, cementum, and alveolar bone, since non-selective osteoinductive or cytotoxic activity at adjacent sites is itself harmful (Sect. 2.2.3). Ligand- or receptor-mediated recognition of resorbing cells, and designs that respond to the acidic pH or elevated enzymatic activity of an inflammatory lesion, have both been proposed [70]. Most of these approaches have been demonstrated only in periodontal or bone-defect models, and whether comparable selectivity can be achieved within the confined root canal and periodontal ligament spaces has not been tested.

#### Retention and release in the oral environment

Salivary flow, enzymatic degradation, and local clearance all limit how long a locally applied material remains at a resorption site. Prolonged retention has been shown experimentally for only a few systems, such as hydrogels that adhere to periodontal tissues [67] and chitosan solutions and derivatives that bind to dentin [71]. For other systems, residence in the root canal has not been measured directly, and persistence in situ is inferred from the in vitro release profile. Release kinetics measured in static buffer do not reproduce the dilution, protein adsorption, and mechanical clearance of the oral cavity. Standardized ex vivo or in situ models that quantify residence time at the root surface are still lacking.

### Nanomaterials for the prevention or treatment of root resorption

Because the studies reviewed below do not report outcomes stratified by the radiographic grade of resorption, nanomaterial-based strategies are discussed in relation to the resorption subtype and etiology rather than to a specific grade. The extent of root loss nevertheless determines whether repair-oriented management remains feasible. Lesions with limited loss are the most plausible candidates for material-based repair, whereas extensive loss with an unfavorable crown-to-root ratio may render the tooth unrestorable irrespective of the material used (Sect.  2.2.2).

#### Inorganic nanomaterials

##### Nano-calcium (Calcium-based nanomaterials)

Calcium is a major mineral component of bone and is required for osteogenesis and tissue remineralization [72]. “Nano-calcium” is used here to denote calcium-based compounds processed to the nanoscale (1–100 nm) rather than elemental calcium. Nanoscale dimensions increase the specific surface area and surface reactivity of these particles, improving their biological availability and their incorporation into mineralized tissues. Three representative materials are considered: Ca(OH)₂ NPs, CaSiO₃ NPs, and nano-hydroxyapatite.

**Ca(OH)₂ NPs**

Calcium hydroxide is widely used as an intracanal medicament for pulpitis and apical periodontitis. Nano-sized formulations have a larger surface area than conventional calcium hydroxide paste and have been reported to penetrate dentinal tubules more deeply and to show greater antibacterial efficacy [73]; no head-to-head clinical comparison in root resorption has yet been performed. These advantages are not consistent, however: deeper tubular penetration has been reported in some studies but not in others, and penetration capacity in anatomically complex canal regions remains limited. Antibacterial activity depends on two synergistic effects: sustained release of OH⁻ ions, which raises the local pH to levels lethal to most endodontic pathogens, and direct interaction of the high-surface-energy particles with bacterial membranes, disrupting them. Ca(OH)₂ NPs show moderate activity against *Enterococcus faec*alis and other endodontic pathogens, although the evidence across studies is heterogeneous [74]. When used for pulpitis, apical periodontitis, or after tooth replantation, these nanoparticles act mainly against infection-associated resorption, and their antibacterial effects have been demonstrated in vitro [74], as have their anti-odontoclastic effects [75]. No preventive effect has been established for resorptions that are not infection-associated, such as ECR, ESR, or OIRR. Their action depends on a high local pH that is not selective for infected tissue and may injure adjacent tissues if the material is extruded beyond the canal. Data on scalable manufacture with a consistent particle size, on long-term biocompatibility, and on the systemic fate of these particles are still lacking.

**CaSiO₃ NPs**

CaSiO₃ nanoparticles combine the ion-releasing bioactivity of calcium-containing materials with the physicochemical stability of silicate-based structures, and have been reported to exert antibacterial effects through reactive oxygen species (ROS)-mediated oxidative stress. Biswas et al. prepared phase-pure α-wollastonite calcium silicate nanoparticles by a low-cost, surfactant-free route with calcination at 850 °C, the lowest temperature reported for this phase [76]. The resulting particles showed increased hardness and Young’s modulus, antibacterial activity against *Streptococcus pyogenes*, *Staphylococcus aureus*, and *Escherichia coli*, and inhibition of biofilm formation [76]. This antibacterial activity was, however, assessed against non-endodontic organisms rather than against endodontic pathogens such as *Enterococcus faecalis*, and the particles have not been tested in root resorption models. Their relevance to IRR therefore remains unestablished. Cytocompatibility was assessed in MC3T3 cells under the tested in vitro conditions, but the particles have not been examined in vivo, and reproducible large-scale production has not been shown.

**Nano-hydroxyapatite (n-HA)**

Hydroxyapatite (HA) is a calcium phosphate–based material widely used as a bone-graft substitute [77, 78]. In its nanoscale form (n-HA), it binds strongly to hard tissue surfaces and can act as a sustained-release carrier for calcium ions (Ca²⁺), providing long-term ion availability for remineralization. It upregulates immunomodulatory and dentin–pulp regeneration genes in dental pulp stem cells [79], and scaffolds seeded with these cells generate mineralized tissue in ectopic implantation models [80], indicating regenerative potential. A commercially available n-HA-containing mouthwash, in which n-HA is combined with other agents, has also shown activity against oral bacteria [81]. Calcium-phosphate/chitosan nanocomplexes similarly reduce bacterial adherence by altering surface charge [82]; this combination of antimicrobial and remineralizing activity distinguishes n-HA from purely antibacterial nanomaterials. Evidence that n-HA repairs resorbed root surfaces in vivo is limited, and its regenerative effect on the root surface has been shown mainly in dentin- and bone-repair models rather than in root-resorption studies. Because n-HA can be osteoinductive, its application to a resorbed root surface also carries the risk of ankylosis and replacement resorption discussed in Sect. 2.2.3.

##### Silver nanoparticles

Silver nanoparticles (AgNPs) and other silver-based materials have been widely investigated for biomedical applications because of their broad antimicrobial activity [83, 84], which is also the property most exploited in dentistry. Their antibacterial efficacy depends largely on how much silver ion (Ag⁺) is released and how fast: smaller particles have higher surface-area-to-volume ratios, release Ag⁺ more rapidly, and therefore show stronger antibacterial effects. Elemental silver is relatively inert, but becomes highly reactive on ionization in the presence of moisture. Antibacterial activity is attributed to binding of Ag⁺ to structural proteins in the bacterial cell wall, which disrupts membrane integrity, alters cellular structure, and ultimately kills the cell [84]. Incorporating silver nanoparticles into dental materials also alters physical properties such as hardness, sorption, solubility, and bond strength, and confers antifungal activity on denture lining materials [83].

Poly(vinyl alcohol) (PVA)-coated silver nanoparticles and farnesol have been tested for their ability to inhibit the growth and adhesion of *Enterococcus faecalis*, *Candida albicans*, and Pseudomonas aeruginosa, and may help control microbial growth in the apical region [85]. In a photothermal approach, AgNPs combined with methylene blue–mediated photothermal treatment eliminated detectable *E. faecalis* colonies in infected root canals ex vivo, whereas laser irradiation alone did not [86]. AgNPs have also been used to modify 17% EDTA (ethylenediaminetetraacetic acid) for root canal irrigation, and the resulting formulation has been shown in vitro to reduce microbial viability and to improve smear-layer removal [87]. Its chemical compatibility has not been fully established, however, because EDTA is a potent chelating agent that may affect the ionic stability, dispersion, and aggregation of AgNPs and the release of silver ions. Direct in-canal evidence remains limited. More broadly, silver nanoparticles raise unresolved safety concerns, including dose-dependent cytotoxicity, oxidative stress, and genotoxicity [88], as well as tissue discoloration and argyria after prolonged exposure [89]. The RR-relevant studies reviewed here did not establish long-term biodistribution or a validated, reproducible manufacturing process for the specific formulations investigated.

##### Bioactive glass

The calcia-phosphosilicate bioactive glass known as 45S5 was introduced by Hench et al. in the early 1970s [90]; the composition most commonly cited for 45S5 is 45 wt% SiO₂, 24.5 wt% Na₂O, 24.5 wt% CaO, and 6 wt% P₂O₅. Its ability to bond to mineralized tissues underpins its use in dental restorative and regenerative materials [91]. In physiological fluids, ion exchange and network dissolution at the surface produce a silica-rich gel layer, on which calcium and phosphate ions accumulate and precipitate as hydroxycarbonate apatite (HCA). HCA resembles the mineral phase of native hard tissues, and the apatite-like interface allows close integration between the material and the surrounding mineralized tissue. Bioactive glass may also support regeneration and microbial control through the release of biologically active ions. Dissolution products containing silicon, calcium, and phosphate have been reported to enhance odontogenic and mineralization-related responses in dental pulp cells, including increased expression of dentin sialophosphoprotein, dentin matrix protein 1, and type I collagen. These findings suggest that the material provides a favorable microenvironment for dentin and bone repair [92].

Bioactive glass has been widely investigated for dentin remineralization [92]. The mechanism depends on the solubility difference between the glass and the surrounding medium: on contact with human plasma or saline solution, mineral precipitation is promoted and HCA crystallizes at the glass–tissue interface. Because the material is hydrophilic, it also draws moisture toward the root canal wall, which has been linked to sealing of the root canal system [92, 93].

Han et al. evaluated an injectable bioactive glass formulation for repairing oral bone defects, and found more favorable defect healing and mineralized tissue regeneration than in hydroxyapatite-treated controls [94]. Lu et al. incorporated the amelogenin-derived peptide (QP5) into mesoporous bioactive glass (MBG) to build a composite delivery system for hard-tissue repair. Compared with MBG or QP5 alone, the composite enhanced dental pulp cell migration and odontogenic differentiation, reflected in higher alkaline phosphatase activity, greater mineralized nodule formation, and upregulation of mineralization-associated genes. It also promoted a dentin-like barrier in a rat direct pulp-capping model. These findings support the potential of bioactive glass–based composites for dentin repair [95]. The evidence nevertheless comes from dentin-repair and pulp-capping models rather than from root-resorption studies, and bioactive-glass compositions that are osteoinductive raise the same concern as for n-HA.

##### Graphene

Graphene is a two-dimensional, atomically thin material composed of sp²-hybridized carbon atoms in a hexagonal honeycomb lattice [96]. Its derivatives vary substantially in lateral size, surface chemistry, oxidation state, and number of stacked layers [96], and include graphene oxide (GO), which occurs as single- or few-layer sheets, reduced graphene oxide (rGO), and graphene nanosheets.

**Fig. 6:**
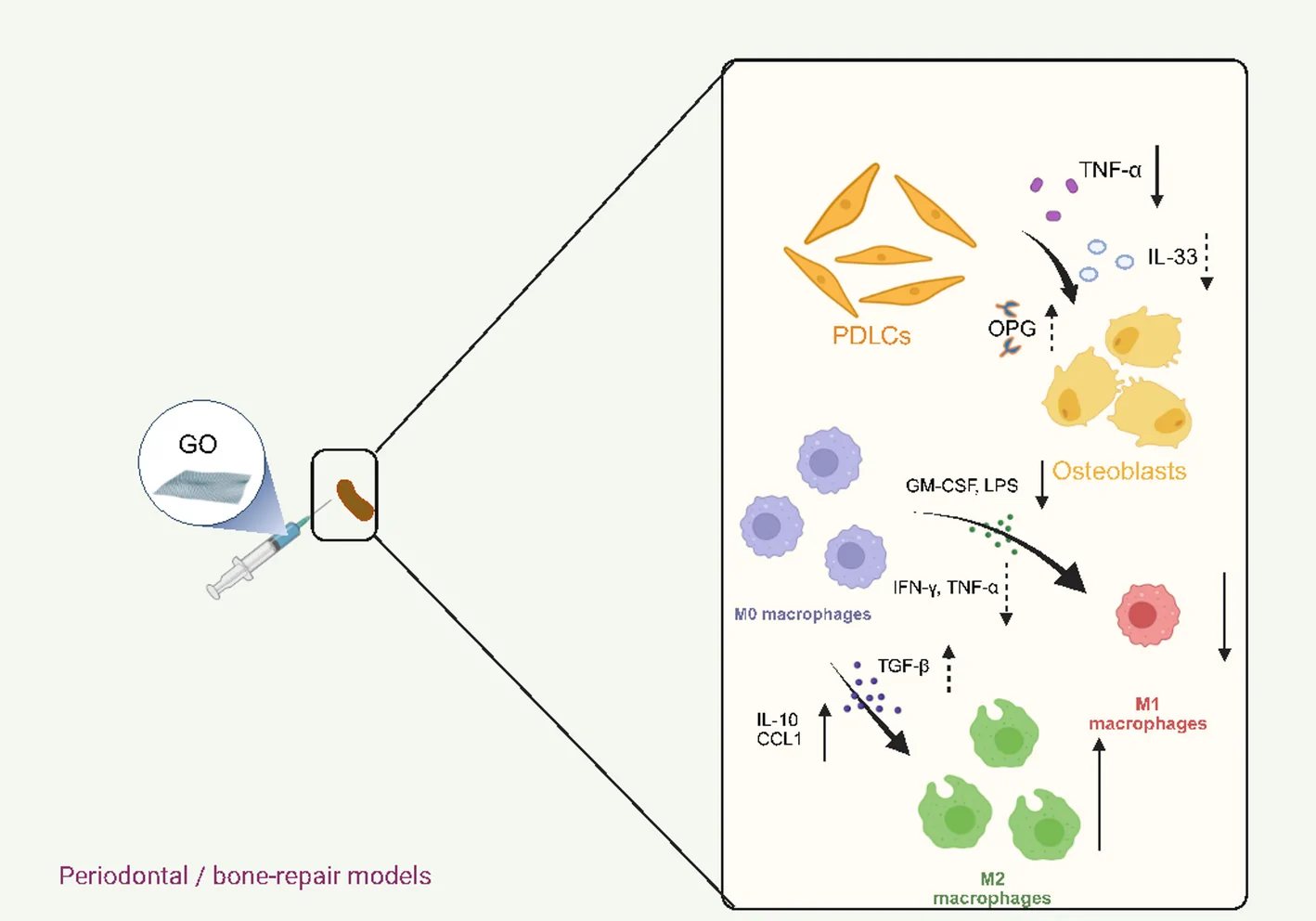
Graphene oxide scaffolds promote PDLC osteogenesis and M2 macrophage polarization. Solid and dashed arrows denote relationships directly demonstrated and proposed, respectively. GO, graphene oxide; PDLCs, periodontal ligament cells; M1 and M2, classically and alternatively activated macrophage phenotypes. Created in https://BioRender.com

Graphene oxide (GO) has attracted considerable interest in biomedical research because its surface chemistry is tunable and it can be incorporated into composite biomaterials [97, 98]. In scaffold systems it improves structural support for cell attachment and growth, and nano-GO–modified titanium surfaces have been shown to modulate osteogenesis-associated signaling pathways, including focal adhesion kinase (FAK)/p38 signaling [99]. In periodontal models, GO-based scaffolds promote osteogenic differentiation of periodontal ligament cells while shifting macrophages toward an anti-inflammatory M2-like phenotype, which favors resolution of resorptive lesions (Fig. 6) [98]. Such evidence comes mainly from periodontal and bone models, whereas rGO has provided direct evidence for root resorption [66]. Under near-infrared irradiation, GO also generates reactive oxygen species and localized hyperthermia that are active against periodontal pathogens [100–102]. This effect is not selective: it may also injure adjacent vital tissues such as the periodontal ligament and pulp, and local temperature control in the oral cavity remains difficult. Although these properties favor periodontal and periradicular regeneration, GO carries the same ankylosis risk described above. Its dose-dependent cytotoxicity in some graphene-based systems [103], together with tissue persistence and limited scalability and reproducibility of manufacture, remains a barrier to clinical translation.

Reduced graphene oxide (rGO) is obtained by partially removing oxygen-containing groups from GO, which markedly changes its surface chemistry and physicochemical behavior. Compared with GO, rGO has higher surface activity and better electronic conductivity, but its lower oxygen content reduces aqueous dispersibility, so surface functionalization is required for biomedical use [104]. rGO is readily functionalized and can carry bioactive cargo: it has been used to load tetrahedral framework nucleic acids, improving their stability and delivery, while suppressing odontoclast activity and supporting osteogenic differentiation [66]. Its high surface activity and efficient drug loading make rGO a candidate carrier for preventing RR, but as with GO, its long-term safety and the risk of ankylosis at the root surface require further evaluation.

##### Mesoporous silica nanoparticles (MSNs)

Silica is abundant in nature, and silica-based nanoparticles are versatile carriers for a wide range of drugs [105]. Their large specific surface area, tunable surface chemistry, and generally favorable biocompatibility have led to their widespread use in dentistry [106], most commonly as reinforcing fillers that improve the mechanical performance and structural stability of restorative materials [107].

According to the nomenclature of the International Union of Pure and Applied Chemistry, porous materials are classified by pore diameter as microporous (< 2 nm), mesoporous (2–50 nm), or macroporous (> 50 nm). MCM-41, first reported in 1992, was the first member of the M41S family of ordered mesoporous molecular sieves [108]; its regular pore structure and tunable channel size subsequently attracted considerable interest. Mesoporous silica nanoparticles (MSNs) are now widely studied as drug-delivery systems because of their high loading capacity, adjustable surface chemistry, controlled degradation, and generally favorable biocompatibility [109].

Talaat et al. evaluated an MSN-based paste in a regenerative endodontic model using immature dog teeth with pulp necrosis, apical periodontitis, and associated root resorption [110]. The paste was placed in the dried root canals and root development was compared with controls; MSN treatment was associated with continued root maturation and repair of the resorptive defects. The study addresses root maturation in immature teeth, a process biologically distinct from the arrest of pathological resorption in mature teeth. In addition, long-term in vivo safety and biodistribution data for MSNs are still lacking, and their scalable manufacture has not been demonstrated.

#### Organic nanomaterials

##### Tetrahedral framework nucleic acid

**Fig. 7:**
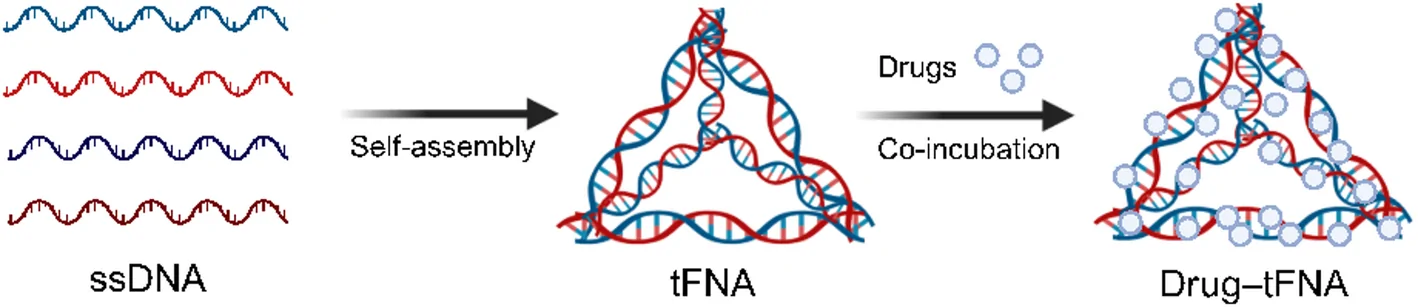
Assembly of ssDNA into tFNA by self-assembly and loading of drugs by co-incubation. ssDNA, single-stranded DNA; tFNA, tetrahedral framework nucleic acid. Created in https://BioRender.com

Seeman et al. first designed the four-arm nucleic acid junction in 1982 [111]; DNA nanostructures have advanced steadily since then and are now widely investigated in biomedical research. Recent advances have enabled the construction of well-defined three-dimensional nanostructures from short nucleic acid strands [112]. Tetrahedral framework nucleic acids (tFNA) are typically assembled from four complementary single-stranded DNA (ssDNA) molecules mixed at an equimolar ratio. Sequence-specific base pairing drives the strands into a rigid tetrahedral architecture with controllable dimensions and edge lengths generally below 10 nm (Fig. 7) [113]. Unlike conventional DNA molecules, tFNA is easy to synthesize, mechanically strong, relatively stable, and readily modified: small molecules, peptides, and nucleic acids can be conjugated to it by electrostatic adsorption, sequence extension, or sequence complementarity, making it a versatile carrier [114]. tFNA has been reported to promote the proliferation and osteo/odontogenic differentiation of dental pulp stem cells through Notch signaling [115]. In lipopolysaccharide (LPS)-stimulated macrophages, tFNA reduced mitogen-activated protein kinase (MAPK) phosphorylation and the production of inflammatory mediators, including nitric oxide (NO), IL-1β, IL-6, and TNF-α, through the modulation of macrophage responses [116]. In experimental periodontitis, these effects were accompanied by reduced alveolar bone loss [117]. Yuan et al. combined tFNA with reduced graphene oxide nanosheets (rGO, described in Sect. 4.2.1.4) to form a delivery complex (tFNA-rGO) [66]. Treatment with this complex reduced orthodontically induced root resorption and odontoclast activity, promoted osteogenic differentiation of periodontal ligament cells (PDLCs), and shifted macrophages toward an M2-like phenotype. As a nucleic-acid nanostructure, however, tFNA also raises concerns about nuclease susceptibility and immune recognition, and its biodistribution, long-term safety, and scalable manufacture remain incompletely defined [118].

##### Chitosan nanoparticles

**Fig. 8:**
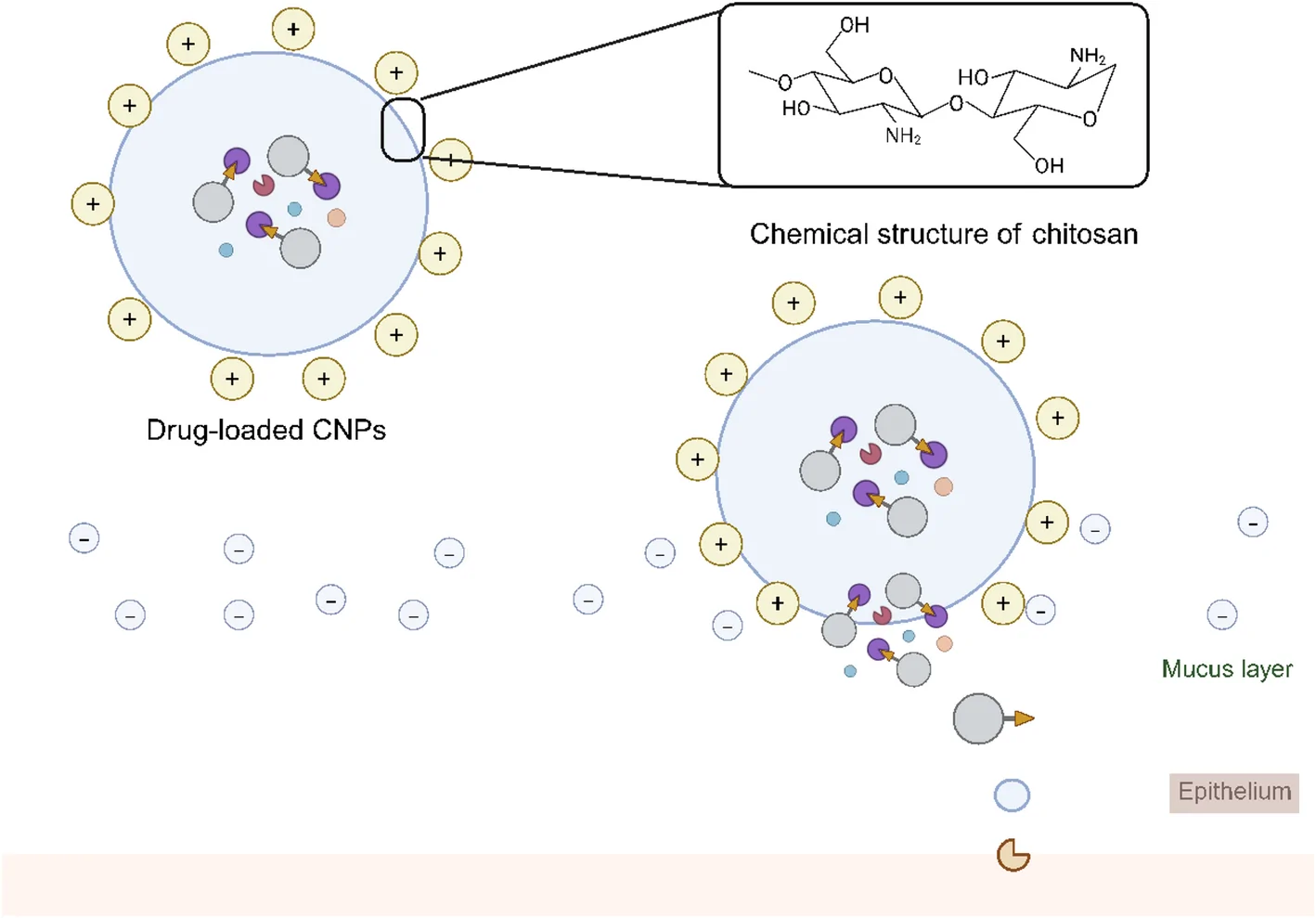
Chemical structure of chitosan and the mucoadhesive mechanism of chitosan nanoparticles as drug carriers. CNPs, chitosan nanoparticles. Created in https://BioRender.com

Chitosan is a biodegradable polysaccharide obtained by alkaline deacetylation of chitin, which is derived mainly from the exoskeletons of crustaceans and insects. Its backbone consists of β-linked D-glucosamine and N-acetyl-D-glucosamine units, whose relative proportions are determined by the degree of deacetylation (Fig. 8). Because it can be prepared as films, gels, and particles, chitosan has attracted considerable attention in biomedical applications [119–121].

Chitosan nanoparticles (CNPs) carry a positive charge on the amino groups of their D-glucosamine units, which interact electrostatically with the negatively charged surfaces of dentin and oral mucosa. This interaction prolongs the residence time of the material and of any agent loaded into it at the site of application (Fig. 8) [122].

Antibacterial activity against *Enterococcus faecalis* depends on the formulation and is greatest for chitosan combined with chlorhexidine [123]; chitosan nanoparticles have also been shown to reduce *E. faecalis* viability on root dentin [124], and their antibacterial activity depends on solubility in acidic environments. When C-2 amino groups are protonated, the positively charged groups interact with anionic components of bacterial membranes, increasing permeability and compromising cellular integrity [121]. Antimicrobial activity has been reported to depend on structural parameters such as the degree of deacetylation, molecular weight, and pattern of chemical substitution [125]. For quaternary chitosan derivatives, however, the relationship between these parameters and activity is not straightforward, and no consistent trend has been observed across the range examined. Chitosan combined with 2% chlorhexidine gel is bactericidal against *E. faecalis* in the root canal [126, 127], and chitosan nanoparticles used as a final irrigant improve apical sealing and push-out bond strength in extracted teeth [128]. These antibacterial properties are relevant to infection-associated resorption such as IRR and EIR.

As drug carriers, CNPs stabilize therapeutic components and facilitate targeted delivery. Yu et al. loaded the extracellular signal-regulated kinase 1/2 (ERK1/2) inhibitor trametinib into a network of catechol-modified chitosan and oxidized dextran to form a composite hydrogel (CCOD-Tra) [67]. After local injection into the periodontal ligament of rats with OIRR, CCOD-Tra adhered to periodontal tissues and released trametinib in a sustained manner. This blocked the ERK1/2 pathway and inhibited osteoclast differentiation in RAW264.7 cells more effectively than trametinib alone [67]. In another study, CNPs were evaluated in a periodontal ligament fibroblast (PDLF)–macrophage co-culture model and in a delayed tooth replantation model in Wistar rats. They suppressed macrophage-derived clastic-cell formation in vitro and reduced root resorption in vivo, supporting their potential as root surface modification agents that promote healing after dental avulsion [129]. Mitochondria-targeted chitosan nanoparticles that modulate the intracellular redox state of periodontal ligament stem cells enhance their osteogenic differentiation under inflammatory conditions [130]. From a translational perspective, CNPs offer favorable biocompatibility and considerable formulation flexibility, but their degradation behavior is difficult to control in the enzyme-rich oral environment and batch-to-batch consistency during large-scale manufacturing has not been achieved.

#### Biological nanoscale systems and therapeutic cargo

##### Extracellular vesicles (EVs)

Extracellular vesicles (EVs) are membrane-enclosed particles whose diameter ranges from about 30 nm to several micrometres, released by a wide range of cell types [131]. EVs mediate intercellular communication, are released by many cell types including mesenchymal stem cells (MSCs), and are present in various body fluids [132]. Compared with MSCs themselves, they may offer practical advantages over viable-cell therapies, although stability, immunogenicity, transport and storage requirements depend on the source, preparation and handling conditions [133], although current isolation methods still give low yield and purity [134]. By delivering bioactive molecules to target cells, EVs participate in tissue regeneration, angiogenesis, immune responses, inflammation, and apoptosis [135].

EV-based approaches have been investigated in preclinical models of root resorption and in adjacent periodontal and bone-repair models. Gandolfi et al. incorporated extracellular vesicles into porous poly(L-lactic acid) scaffolds containing dicalcium phosphate dihydrate and calcium silicate. The constructs increased expression of osteogenic markers, including osteocalcin, osteopontin, osteonectin, and type I collagen, indicating potential value for periodontal regeneration [136]. This evidence is nevertheless limited to in vitro culture of human adipose-derived mesenchymal stem cells, and the authors called for additional animal-model investigations. Two further studies provide direct RR evidence. In orthodontically induced root resorption, human dental follicle stem cell–derived extracellular vesicles (DFSC-Exos) reduced PDLC pyroptosis through miR-140-3p–mediated regulation of the DNMT1/SOCS1/NF-κB pathway, and this was accompanied by reduced M1-like macrophage polarization and odontoclast formation, attenuating root resorption [137]. In a rat model of delayed maxillary first molar replantation, periodontal injection of extracellular vesicles from healthy human periodontal ligament fibroblasts reduced resorption lacunae and the number of TRAP-positive odontoclasts. Treatment was associated with increased OPG expression in the periodontal ligament and improved periodontal tissue repair [138].

These findings indicate biological activity rather than established clinical efficacy, because the reductions in PDLC pyroptosis, M1-like macrophage polarization, and odontoclast formation have been observed only in preclinical models. Translation is further constrained by the limited availability of techniques for producing and isolating extracellular vesicles with high purity and yield, by the often substantial cost of cell culture, and by unresolved questions about long-term safety. Heterogeneity, batch-to-batch variability, and classification as biological products further complicate clinical development.

##### MicroRNAs

MicroRNAs (miRNAs) are short non-coding RNAs that regulate the balance between bone formation and resorption and influence the fate of cementoblasts; because they are rapidly degraded in biological fluids, they require delivery by extracellular vesicles or nanocarriers. Several miRNAs have shown anti-resorptive effects in root resorption models. miR-155-5p inhibits odontoclast differentiation by targeting CXCR2 [139], and the Runx1/miR-26a/Jagged1 axis controls odontoclastogenesis and alleviates OIRR [140]. miR-223-3p mitigates mitochondrial dysfunction and cementoblast apoptosis [32], and EV-delivered miR-140-3p inhibits pyroptosis in PDLCs and reduces root resorption [137]. The evidence is nevertheless derived mainly from in vitro and animal studies and from human observational expression data, and the delivery efficiency, off-target effects, and dose-response relationships of miRNA-based strategies remain poorly defined.

##### Growth factors and peptides

Growth factors and peptides can promote periodontal and cementum repair, but their short biological half-lives motivate investigation of local or sustained-release delivery. Transforming growth factor-β (TGF-β) signaling upregulates osteoprotegerin in cementoblasts and thereby inhibits odontoclastogenesis [141], and platelet-derived growth factor-BB (PDGF-BB) has been reported to modulate root resorption after tooth replantation [142]. Their clinical use nevertheless raises additional concerns, including off-target effects and the potential to promote aberrant cell proliferation, so their root-resorption-specific efficacy and safety require dedicated evaluation. Table 1 summarizes the function category, mechanism, application, indications, and limitations of each nanomaterial.

**Table 1:** Nanomaterials for root resorption: functions, forms of application, indications, and limitations

Nanomaterial	Function category	Mechanism	Application	Indication	Limitations	Refs
Inorganic Nanomaterials						
**Ca(OH)₂ nanoparticles**	Antimicrobial activity; anti-odontoclastic activity	Sustained OH⁻ release raising local pH; membrane disruption.	Intracanal medicament (nanoparticle suspension or paste)	Infection-associated IRR and EIR (pulpitis, apical periodontitis, or teeth after replantation); in vitro evidence only	Non-selective action (high local pH); extrusion injury; no RR endpoints	[74, 75]
**Calcium silicate (CaSiO₃)**	Antimicrobial activity	ROS-mediated oxidative stress.	Bioactive antibacterial filler candidate for endodontic and bone materials	No RR indication established; antibacterial activity reported in vitro against non-endodontic organisms only	Non-endodontic organisms only; no in vivo data	[76]
**Nano-hydroxyapatite (n-HA)**	Remineralization; regeneration; antimicrobial activity	Ca²⁺ release and binding to hard tissue; upregulation of dentin–pulp regeneration genes.	Bone-graft substitute, remineralizing agent, oral-care formulations and scaffolds for dental pulp stem cells	Root-surface and cementum repair after resorption; dentin remineralization	Ankylosis risk; cementum-PDL interface untested	[79–81]
**Silver nanoparticles (AgNPs)**	Antimicrobial activity	Ag⁺ release; binding to cell-wall proteins and membrane disruption.	17% EDTA irrigant modification, intracanal medicament, sealer additive and photothermal irrigation	Infection-associated IRR and EIR; caries (other indication)	Cytotoxicity; discoloration; EDTA compatibility; biodistribution untested	[83–89]
**Bioactive glass**	Remineralization; regeneration; antimicrobial activity; sealing	Ion release and hydroxycarbonate apatite precipitation; odontogenic gene upregulation.	Remineralizing and sealing materials, endodontic sealers, direct pulp-capping and regenerative endodontic composites	Dentin remineralization, root canal sealing, dentin and bone defect repair; root-surface repair proposed	Ankylosis risk; ectopic mineralization untested	[92, 93, 95]
**Graphene oxide (GO)**	Anti-inflammatory / immunomodulatory activity; regeneration	Photothermal effect; M2 macrophage shift; osteogenic differentiation.	Periodontal scaffold or coating, implant abutment surface modification and near-infrared photothermal disinfection	Periodontal and periradicular regeneration; anti-inflammatory M2 shift in periodontal models; proposed for OIRR and ESR	Non-selective heating; cytotoxicity; persistence; ankylosis risk	[96–101]
**Reduced graphene oxide (rGO)**	Anti-odontoclastic activity; delivery; regeneration	Suppression of odontoclast activity; osteogenic differentiation; nanocarrier for tFNA.	Nanocarrier for tetrahedral framework nucleic acid delivery by periodontal ligament injection	OIRR; reduced root resorption and odontoclast activity in a mouse model	Ankylosis risk; batch consistency	[66]
**Mesoporous silica nanoparticles (MSNs)**	Regeneration; delivery	High loading capacity with controlled release; root maturation in immature teeth.	Drug-delivery carrier; intracanal paste in regenerative endodontic procedures	Root maturation in immature teeth with pulp necrosis and apical periodontitis	Biodistribution; mature-tooth endpoints untested	[110]
**Organic Nanomaterials**						
**Tetrahedral framework nucleic acids (tFNA)**	Anti-inflammatory / immunomodulatory activity; delivery	MAPK pathway downregulation; reduced NO, IL-1β, IL-6 and TNF-α; M2 shift.	Drug and nucleic-acid carrier; periodontal ligament injection as the tFNA-rGO complex	Anti-inflammatory and immunomodulatory action in OIRR	Immunogenicity; nuclease stability; scale-up	[114–117]
**Chitosan nanoparticles (CNPs)**	Antimicrobial activity; delivery	Membrane disruption by protonated amino groups; ERK1/2 pathway inhibition; mucoadhesion.	Irrigant, intracanal medicament (with 2% chlorhexidine), mucoadhesive carrier, injectable periodontal hydrogel (CCOD-Tra) and root-surface modifier	Infection-associated IRR and EIR; OIRR (trametinib-loaded hydrogel); root resorption after delayed replantation	No RR-specific evidence for the antimicrobial and adhesive formulations; batch-to-batch consistency	[67, 71, 122, 124], 126– [129, 143]
**Biological Nanoscale Systems and Therapeutic Cargo**						
**Extracellular vesicles (EVs)**	Anti-inflammatory / immunomodulatory activity	miR-140-3p–mediated inhibition of PDLC pyroptosis; OPG upregulation.	Periodontal ligament injection; enrichment of scaffolds for periodontal regeneration	OIRR and root resorption after delayed replantation	Purity and yield; heterogeneity; biological-product status	[137, 138]
**MicroRNAs (miRNAs)**	Anti-odontoclastic activity	miR-155-5p targeting CXCR2; Runx1/miR-26a/Jagged1 axis; miR-223-3p–mediated protection of cementoblasts.	Therapeutic cargo delivered by extracellular vesicles or nanocarriers (no product)	OIRR and odontoclastogenesis	Delivery; off-target effects; dose	[32, 139, 140]
**Growth factors and peptides (TGF-β**,** PDGF-BB)**	Regeneration	TGF-β–induced OPG upregulation in cementoblasts; PDGF-BB modulation after replantation.	Sustained-release delivery systems for periodontal and cementum repair	Root-surface repair and replantation; cementoblast OPG upregulation and post-replantation modulation	Short half-life; off-target proliferation; RR safety untested	[141, 142]

## Discussion

The available evidence is unevenly distributed across materials and RR subtypes: most reports come from in vitro studies or animal models, and only a few materials have been evaluated directly in root-resorption models. Safety, physicochemical characterization, targeting specificity, retention in the oral environment, and scalable manufacture are requirements shared across material classes, and each needs direct RR evidence before clinical evaluation can be justified.

As nanotechnology advances, an increasing array of nanomaterials is being developed for clinical use, and concerns about their safety have grown in parallel. Because these materials are intended for the oral environment—a site of continuous salivary flow, enzymatic activity, and intimate contact with mucosal and periodontal tissues—their long-term residence and potential for systemic absorption warrant careful evaluation. Their nanoscale dimensions may also allow them to cross biological barriers and reach compartments that conventional formulations access poorly, raising concerns about unintended systemic exposure and long-term toxicity. Specific safety concerns have already been identified for silver, graphene-based materials, and nucleic-acid-based systems, and these require dedicated assessment rather than being deferred. Most available biocompatibility data come from simplified in vitro models using conventional cell lines and dispersed nanoparticles. These systems do not fully reproduce the cellular diversity, immune responses, and multicellular interactions present in vivo and therefore limit translation to actual tissue behavior [144]. More physiologically relevant in vivo models, together with systematic approaches to improving biocompatibility, are needed to define the biological effects and safety profiles of these materials.

Nanomaterials applied for RR act on more than one cell population. Periodontal ligament cells and their stem cells, cementoblasts, odontoblasts, pulp cells, osteoclasts, osteoblasts, macrophages, and the oral microbiota are all potentially exposed, and the net therapeutic effect depends on the balance between resorptive and reparative responses. In the studies reviewed here, cytocompatibility was usually assessed in only one or two cell types, most often fibroblasts or osteoblast-like lines and rarely cementoblasts or odontoblasts, which are the cells most directly relevant to root-surface repair. Systematic evaluation across the cell populations present at the resorption site, ideally in co-culture or ex vivo tissue models, would provide a more realistic safety profile than single-cell-type assays.

For antimicrobial nanomaterials, repeated or prolonged exposure raises a further concern that has received little attention in the RR literature. Silver, chitosan, and calcium hydroxide act through largely non-specific mechanisms, and reduced bacterial sensitivity to such agents was long considered unlikely. Experimental adaptation to silver and metal-oxide nanomaterials has since been documented, involving efflux systems, biofilm formation, and cell-envelope changes that overlap with the mechanisms of antibiotic resistance [145]. Because intracanal medicaments and irrigants are delivered repeatedly in clinical endodontics, whether nanomaterial-based antimicrobials perturb the oral microbial community or induce cross-tolerance to conventional antimicrobials warrants explicit investigation.

Beyond safety, scalable and reproducible production is a prerequisite for clinical application. At present, however, the synthesis of many candidate nanomaterials remains complex, time-intensive, and costly, and reproducible manufacture is among the validation needs listed in Table 2 and has impeded their progression from the laboratory to the clinic [146]. Future research should therefore prioritize strategies that preserve the advantageous properties of these materials while simplifying fabrication, exploring combinations with conventional materials, and reducing production costs without compromising therapeutic efficacy. These translational challenges must be resolved before nanotechnology-based strategies can be reliably integrated into the prevention and clinical management of root resorption.

**Table 2:** Development stage, human evidence from other indications, and level of evidence for each material or intervention

Material or specific intervention	RR-specific development stage	Human evidence from other indications	Key validation steps	Evidence level	Refs
**Ca(OH)₂/poly(lactic-co-glycolic acid) (PLGA) nanoparticles (intracanal)**	Mechanistic evidence; direct RR efficacy not established	Conventional intracanal use; evidence from a different formulation	RR-efficacy endpoints; exposure to the exact formulation	SR/MA (low-moderate certainty); IV-D	[74, 75]
**CaSiO₃ nanoparticles**	Non-RR assays only	Non-endodontic in vitro data; no human data	RR endpoints; in vivo tolerability	IV-D	[76]
**n-HA formulations**	Non-RR evidence; no direct RR repair demonstrated	Marketed oral-care products; evidence from different formulations	Cementum-PDL integration endpoints; dose-response	IV-D; SA (mouse)	[79–81]
**AgNP formulations**	Non-RR evidence for candidate intracanal formulations	Nanosilver-fluoride caries randomized controlled trial (RCT); evidence from a different indication	Formulation-specific testing; RR endpoints	EX; IV-D; HI-other	[83– [89, 147]
**Bioactive-glass-containing composites**	Non-RR evidence for the nanoscale composites	Clinical dental glass materials; evidence from different formulations	Particle-size and cargo effects; PDL integrity; ectopic mineralization	IV-D; SA (rat)	[92, 93, 95]
**GO composites/coatings**	Non-RR evidence; RR application not yet evaluated	No human data for graphene-based RR candidates	Root-specific efficacy; photothermal dose; clearance	IV-D; SA (rat)	[96–101]
**tFNA-rGO complex**	Direct RR evidence of therapeutic effect	Rodent OIRR only; no human data	PDL preservation; dose-response; GMP compliance	SA-RR (mouse)	[66]
**MSN scaffolds with/without bone morphogenetic protein-2 (BMP-2)**	RR-related findings	Dog regenerative endodontics only; no human data	Mature-tooth validation; RR endpoints; longer follow-up	LA (dog)	[110]
**Standalone tFNA**	Cell-level and non-RR evidence	Periodontal models only; no human data	Structure-activity testing; exposure; quality control	IV; SA (rat)	[114–117]
**Antimicrobial/adhesive CNPs**	Non-RR evidence for these formulations	Intracanal chitosan clinical data; antimicrobial outcomes	Nanoparticle characterization; antimicrobial, sealing and RR outcomes	EX; IV-D; HI-other	[122, 124], 126– [128, 143]
**Engineered drug/photosensitizer CNPs**	Direct RR evidence of efficacy	Rat delayed replantation only; no human data	Ankylosis outcomes; local retention; release kinetics	IV; SA-RR (rat)	[129]
**Dental-cell-derived EV preparations**	Direct RR evidence of biological activity	Human cell source only; no human RR data	Identity and potency assays; stability testing	IV-RR; SA-RR (rat)	[137, 138]
**miRNAs as cargo/targets**	Therapeutic and target-level evidence	No clinical product	Delivery system; dose; formulation-specific efficacy	IV; SA-RR (rat)	[32, 139, 140]
**TGF-β**	Direct RR evidence; biological intervention	Mouse OIRR only; no human data	Formulation; therapeutic window	IV; SA-RR (mouse)	[141]
**PDGF-BB**	Direct RR evidence; biological intervention	Periodontal clinical use; evidence from a different formulation	Replantation outcomes; human RR efficacy/safety	LA (dog)	[142]
**CCOD-Tra hydrogel**	Direct RR evidence; polymeric delivery system	Rat OIRR only; no human data	Local drug exposure; physiological remodeling; carrier safety	IV; SA-RR (rat)	[67]

Note. Evidence level: IV, in vitro; IV-RR, in vitro—RR-specific; IV-D, in vitro—non-RR dental model; EX, ex vivo; SA, small-animal; SA-RR, small-animal RR model; LA, large-animal; HI-other, human interventional evidence (other indication); SR/MA, systematic review or meta-analysis. Other abbreviations: GMP, good manufacturing practice. Development-stage qualifiers: direct RR, outcome measured in a root-resorption model; RR-related, RR findings secondary to another endodontic outcome; non-RR, models outside RR; mechanistic, mechanism only; target-level, cargo or target without a delivery product. Codes other than HI-other denote preclinical evidence (cell, ex vivo or animal studies)

Most research on these nanomaterials has focused on repairing and preventing defects in bone or coronal hard tissue, and comparatively few studies have examined nanomaterials specifically for the prevention and clinical management of RR. As discussed in Sect.  3, strategies developed for bone resorption are not directly transferable to the root: bone undergoes continuous physiological remodeling through coupled osteoclast and osteoblast activity, whereas dentin does not remodel and cementum lacks such coupled remodeling. Osteoinductive nanomaterials applied to a resorbed root surface may in particular induce dentoalveolar ankylosis and replacement resorption, so root-surface repair should favor cementum-like (cementogenic) rather than bone-like mineralized tissue. Materials adapted from bone research should therefore be treated as a source of mechanistic hypotheses requiring root-resorption- and cementum-specific validation, not as a directly transferable strategy. Whether MSNs functionalized to target cementum or dentin, with drug-loading strategies tailored to the root surface microenvironment, can improve outcomes in RR remains an open question.

Most current evidence for nanomaterial-based strategies in RR remains preclinical, and substantial challenges related to efficacy, safety, targeting, and manufacturing must be addressed before clinical translation. A structured translational pathway is therefore needed. It should proceed from material selection and physicochemical characterization, through RR-specific in vitro models, dentin/cementum interaction and odontoclast assays, periodontal ligament models, and small- and large-animal RR models, to pharmacokinetic, biodistribution, and toxicological assessment and manufacturing under Good Manufacturing Practice (GMP) before clinical trials. Dentoalveolar ankylosis should be included as a dedicated safety endpoint during preclinical evaluation.

## Conclusions

Root resorption arises from clastic-cell activity that is normally restrained by the unmineralized tissues covering the root surface, and its management remains limited to removing the causative stimulus, disinfecting and sealing the affected root, and monitoring the lesion. These approaches cannot restore lost cementum or dentin, and lesions of infectious, mechanical, or traumatic origin differ sufficiently in their biological requirements that no single strategy is applicable to all of them. Nanomaterials offer several advantages for this clinical problem, including localized delivery with improved retention, controlled release, antimicrobial activity, and modulation of the inflammatory and odontoclastogenic environment, and they therefore represent a rational direction for the development of subtype-specific therapies. The evidence supporting these applications nevertheless remains predominantly preclinical. Direct evidence from root-resorption models is available for only a small number of materials; most reports derive from periodontal, endodontic, or bone models, and no nanomaterial has yet reached root-resorption-specific clinical use. Safety and biocompatibility, targeting specificity, retention in the oral environment, and scalable and reproducible manufacture remain unresolved, and the risk of dentoalveolar ankylosis requires particular attention when osteoinductive materials are applied to a resorbed root surface. Progress will depend on characterizing candidate materials physicochemically, evaluating them in root-resorption-specific models, and defining their long-term behavior and safety, and it will require concerted interdisciplinary effort between materials science and clinical dentistry.

## Data Availability

No datasets were generated or analysed during the current study.
